# Dr. J. G. Douglas Kerr

**Published:** 1906-12

**Authors:** 


### Dr. J. G. DOUGLAS KERR, Bath.

By the death of J. G. Douglas Kerr, on November 14th, Bath lost one of her best friends, for there can be no doubt that his personality and the great confidence he instilled in his patients brought to the city of Bath from all quarters, and especially from north of the Tweed, patients of wealth and influence who had never before sought assistance from the Bath waters.

He was educated at Glasgow University, where he had a distinguished career, taking among other honours the gold medal in Surgery. He graduated in the year 1880, and after holding the appointment of house surgeon to the Glasgow Infirmary and a short bout of travelling, he came to Bath as partner to the late Mr. T. G. Stockwell. He soon made up his mind to perfect himself in all matters appertaining to the thermal treatment of disease, and almost immediately he had done so he developed a practice which increased by leaps and bounds. It was in the late autumn of 1905 that a severe attack of influenza played sad havoc with a heart overstrained by his hard-working life, and which probably had been overtaxed during those years in which he spent his holidays in climbing the high mountain peaks of Switzerland and the Austrian Tyrol. Directly he was well enough to travel he started for the Canary Islands, but before he reached the Bay another attack of influenza almost proved fatal. A sojourn in Orotava of several months greatly improved his condition; and after a few days at home he went to Nauheim, where for six weeks he underwent the treatment. A short stay in Switzerland seemed to do him much good, and in the early part of September he came home and resumed some of his work, when it was hoped by his many friends that his heart had rallied from its serious condition. On November 12th another attack of his enemy again laid him low, and in spite of all that human aid could do the end came on November 14th.

About seven years ago he took to yachting as a pastime, and for the first three years cruised in the well-known old racer *The Raven;* but he soon decided to join the ranks of the racing yachtsmen, and commissioned W. Fife to build him the 106-ton yawl *The Valdora.* During her first year she won no less than twenty-three flags in high-class handicap racing, while in her second year she won in record time the International Cup from Dover to Heligoland, given by His Majesty the German Emperor, besides twenty other winning flags. During the year 1905 she was not so successful, being heavily handicapped, but she won the Royal Albert Cup, which she had lost the previous year by only a few seconds.

Dr. Kerr was unmarried, but, in addition to a large circle of friends and patients, has left to mourn his loss a mother, to whom no son was ever more devoted, and to her the profession of Bath extend their sincerest sympathy.

On November 19th he was laid to his last long rest in Bathwick Cemetery, amidst manifestations of regard and deep regret. His methodical and co-ordinate mind, his untiring energy, his whole-hearted interest in those who consulted him soon gained for him their confidence and their appreciation of his opinion and advice, and again and again they visited Bath to seek his aid. But it was something more than mere confidence in his opinion and the value of his advice that had so rapidly increased his large connection and held it together with no uncertain hand. The hundreds of letters now on his table bear overwhelming testimony to the real, affectionate esteem in which he was held by all his patients and disclosed the secret of his great success.

For this wonderful personality and popularity he has had to pay the penalty, and there can be no doubt that the long hours of strenuous work for many months in the year told upon what was never a strong constitution, and his sorrowing relatives, his numerous friends and patients, and the city of Bath mourn the loss of one whose life's record, short as that useful life has been, will ever be held in affectionate remembrance.

"To esteem, to know, to love, and then to part,
Makes up life's tale to many a feeling heart."

